# Effects of Different Fermentation and Clarification Methods on the Color, Physicochemical Characteristics, and Aroma Profile of Healthcare Cornus–Kiwifruit Composite Wine

**DOI:** 10.3390/foods14101705

**Published:** 2025-05-11

**Authors:** Cuiyan Zeng, Xueru Zhang, Junxia Zhang, Shuiyan Pan, Keqin Chen, Yulin Fang

**Affiliations:** College of Enology, Northwest A&F University, Yangling 712100, China; cyaan@nwafu.edu.cn (C.Z.); zhangxueru@nwafu.edu.cn (X.Z.); junxia@liverpool.ac.uk (J.Z.); psy19977448177@nwafu.edu.cn (S.P.)

**Keywords:** kiwifruit, Cornus officinalis, composite fruit wine, fermentation

## Abstract

A lack of distinctive features has become a significant factor limiting the development of kiwi wine. However, the rapidly growing trend of healthcare-oriented composite fruit wine with health functions and diverse flavors presents a way to address this issue. A kiwi wine fermentation method was investigated by incorporating the medicinal and edible fruits of Cornus officinalis. The results indicate that adding Cornus officinalis introduced a unique component known as iridoid glycosides to the wine. Additionally, the concentrations of phenols, total iridoid glycosides, and most aroma compounds in the wine increased after the addition of crushed Cornus officinalis following alcoholic fermentation. As the proportion of Cornus officinalis in the kiwi wine rose, so did polyphenolic substances and total iridoid glycosides; however, this diminished the wine’s clarity. Additionally, a yeast addition of 200 mg/L demonstrated optimal fermentation capabilities, and a bentonite addition of 1.1 g/L exhibited an outstanding clarifying effect. These results not only enhance nutritional value and quality but also provide a theoretical foundation for the production of high-quality Cornus–kiwifruit composite wine.

## 1. Introduction

Kiwifruit has become an indispensable and commercialized product in the international market [1,2]. It is rich in a wide array of nutrients, such as vitamin C, organic acids, minerals, proteins, carbohydrates, phenols, and other bioactive compounds, as well as trace elements [3,4,5,6]. These components endow kiwifruit with high nutritional and medicinal value, including antioxidant and anti-cancer benefits, antibacterial properties, and relief from constipation [7,8,9]. The expansion of kiwifruit cultivation has significantly increased its production. However, numerous problems have arisen in the industry, such as oversupply and unmarketability issues [10,11]. Thus, converting kiwifruit into composite wine not only mitigates economic losses for orchards by fully utilizing the fruit, but also enhances its nutritional value and bioactive properties while preserving its inherent nutritional characteristics and varietal qualities [12,13,14].

There are still limitations in the development of kiwi wine, including a lack of aroma and distinctive characteristics [15]. Healthcare composite fruit wine offers an effective solution to these issues, enhancing the wine with diverse aromatic qualities and significantly improving its health benefits [16]. For instance, incorporating cyclocarya into kiwifruit wine and adding blueberries to red wine can enhance both the nutritional value and aromatic components [17,18]. The healthcare composite wine approach has emerged as a prominent direction for future research and development in kiwi wine production. Cornus officinalis is a widely used medicinal herb in Asia, with notable medicinal properties [19,20], and it contains various active compounds, including flavonoids, as well as iridoids and their glycosides [21,22,23]. Pharmacological studies have demonstrated that Cornus officinalis has numerous bioactive functions, including anti-inflammatory, antioxidant, and anti-cancer properties, as well as benefits in lowering blood sugar and blood pressure, protecting nerves, enhancing retinal function, and treating lipid metabolism disorders [24,25,26,27,28,29,30]. The economic potential of Cornus officinalis thus extends to medical applications, food products, and horticulture. Research has primarily focused on its mechanism of action and functional activity, such as its liver and kidney tonifying effects, hypoglycemic activity, and protective influence on hippocampal neurons [31,32,33].

Incorporating Cornus officinalis into kiwi wine introduces new bioactive compounds, such as the iridoid glycosides [34], but there is a lack of research on the optimal fermentation methods for Cornus–kiwifruit wine. This study investigates the effects of varying ratios of kiwi and Cornus officinalis, different forms and timing for Cornus officinalis addition, the quantity of yeast added during fermentation, and the amount of bentonite used during clarification. The impact of these factors on the basic physicochemical parameters, polyphenolic compounds, iridoids, volatile substances, color, and clarity of Cornus–kiwifruit wine was assessed, with the results providing a theoretical foundation for the production of Cornus–kiwifruit wine with enhanced nutritional and health features.

## 2. Materials and Methods

### 2.1. Samples

‘Xuxiang’ kiwifruits and Cornus officinalis were utilized to produce Cornus–kiwifruit wine and to examine the impact of different treatments on its characteristics. Kiwifruits were sourced from local orchards in Yangling, Shaanxi Province, China (N 34°14′ to 34°20′, E 107°59′ to 108°08′). Fruits were harvested in four batches at commercial maturity, determined by the following ripening indices: soluble solids content (SSC) of 15–16°Brix and titratable acidity (TA) of 12–13 g/L. The Cornus officinalis fruits were obtained from farmers and collected at their commercial ripening stage in Hanzhong, Shaanxi Province, China (N 32°08′ to 33°52′, E 105°29′ to 108°16′). Dried whole kiwifruits and Cornus officinalis fruits were carefully selected, uniformly crushed using a mechanical crusher, treated with sulfur dioxide (SO_2_, 60 mg/L; analytical grade; Tianjin Tianli Chemical Reagent Co., Ltd., Tianjin, China), and added to the must. The must was prepared by blending Cornus officinalis and kiwifruit in predetermined ratios. Pectinase treatment was performed using 20 mg/L LALLZYME EX pectinase (LALLEMAND, Blagnac, Haute-Garonne, France) to enhance juice extraction. Sucrose was added to adjust the soluble solids content (SSC) to 20°Brix. Alcohol fermentation was initiated through inoculation with varying concentrations of CVE-7 yeast (Angel Yeast Co., Yichang, Sichuan, China) and maintained at controlled temperatures of 16–18 °C. Fermentation was terminated by adding 50 mg/L sulfur dioxide when the specific gravity reached 0.985–0.995. The wine samples were clarified through filtration by adding bentonite (Sheng Feng Agriculture and Animal Husbandry Co., LTD., Fushun, Liaoning, China) at varying concentrations.

The experimental design involved a systematic evaluation of Cornus officinalis fruit incorporation in wine production through four distinct methodological approaches: whole fruit addition prior to fermentation (WBF), whole fruit addition post-fermentation (WAF), crushed fruit addition before fermentation (CBF), and crushed fruit addition after fermentation (CAF). Using a systematic comparative analysis, we established the core processing parameters for Cornus–kiwifruit composite wine. Optimization experiments examined the influence of three critical processing variables and their impacts on key oenological characteristics, including varying Cornus officinalis to kiwifruit mass ratios (1:20, 1:30, 1:40, and 1:50), different yeast inoculation concentrations (100, 150, 200, and 250 mg/L), and a range of bentonite fining levels (0.8, 0.9, 1.0, 1.1, and 1.2 g/L). The experimental methodology is detailed in Table S1, which specifies the Cornus officinalis incorporation method, the Cornus officinalis to kiwifruit mass ratio, the yeast inoculum concentration, and the bentonite dosage for clarification.

### 2.2. Detection of Physicochemical Parameters

A handheld refractometer (ATAGO, Guangzhou, Guangdong, China) was utilized to determine the soluble solids content, while a pH meter (Mettler Toledo, Changzhou, Jiangsu, China) was used to assess the pH of the wine [35]. The direct titration method was employed to evaluate both reducing sugar and total acidity [36,37]. Additionally, the alcohol content was determined using the distillation method [38]. Each measurement was repeated three times for each wine sample.

### 2.3. Determination of Polyphenolic Substances and Iridoid Glycosides in Cornus-Kiwi Fruit Wine

Iridoids and their glycosides are the most abundant compounds in Cornus officinalis fruit and represent unique components of this plant, possessing numerous significant functions. The total iridoid glycoside content was determined using photometric analytical methods, with the results expressed as rutin [39]. The absorbance of iridoid glycosides was measured at 236 nm using an Agilent Cary 60 UV-VIS spectrophotometer (Agilent, Palo Alto, CA, USA), and the total content was calculated based on the standard curve. The measurements were repeated three times for each wine.

The total anthocyanin content in the Cornus–kiwifruit wine was determined using the pH differential method, with the final results expressed as malvidin [40]. Additionally, the NaNO_2_-AlCl_3_ method was employed to measure the total flavonoid content, expressed as rutin [41]. The total phenolic content in the composite fruit wine was assessed using an Agilent Cary 60 UV-VIS spectrophotometer (Agilent, Palo Alto, CA, USA) according to the Folin-Ciocalteu assay, with the results expressed as gallic acid [42]. Each index of the wine samples was evaluated in triplicate.

### 2.4. GC-MS Analyses of Volatile Compounds in Wines

Volatile compounds were extracted using headspace solid-phase microextraction (HS-SPME) and analyzed using gas chromatography–mass spectrometry (GC-MS). The wines were analyzed directly. A solution was prepared by adding 1.00 g of NaCl (analytically pure, Sinopharm Chemical Reagent Co., Ltd., Shanghai, China.) and 5 mL of wine to a 15 mL sample bottle, followed by a 1 cm magnetic stir bar and 10 μL of 4-Methyl-2-pentanol (Wuhan UCchem Biotechnology Co., Ltd., Wuhan, Hubei, China). An automatic sampler was used to extract the volatile compounds in the wine, and the sample bottle was then placed on a magnetic heating stirring table at 40 °C for 30 min to ensure temperature equilibrium. The activated extraction head was inserted into the sample’s headspace and operated at 40 °C for an additional 30 min. Subsequently, the extraction head was removed and placed in the GC injection port for analysis, which lasted 8 min. An Agilent gas chromatography (GC) model 7890 instrument was employed for the analysis, integrated with an Agilent 5975 mass spectrometer (MS), a 7683 automatic sampler (Agilent, Palo Alto, CA, USA), and a DB-Wax capillary column (60 m × 0.25 mm × 0.25 µm; Agilent, Palo Alto, CA, USA). The injection port was maintained at 250 °C. Initially, the column temperature was 50 °C and held constant for 5 min. Qualitative analysis was performed by comparing retention indices established using n-alkanes (C7-C24) (Supelco, Bellefonte, PA, USA) and matching mass spectra with those of certified reference standards. All spectral data were verified against the NIST 11 mass spectral database (National Institute of Standards and Technology). For quantitative analysis, 4-Methyl-2-pentanol was employed as an internal standard with calibration performed via a five-point calibration curve methodology, with calculations conducted following the curve established in our laboratory’s prior work [43]. Final results were reported as concentration values (μg/L) in the respective wine samples. Three technical replicates were analyzed per sample.

The odor activity values (OAVs) of individual compounds were calculated by dividing their concentrations in wine samples by their odor thresholds in water. Only aroma-active compounds with OAVs > 1, indicating a direct contribution to the aroma, were selected for further analysis. The odor threshold values were obtained from the literature [44].

### 2.5. Clarity and Color Analysis

The clarity of the wine samples was determined using an Agilent Cary 60 UV-VIS spectrophotometer (Agilent, Palo Alto, CA, USA) [45]. The upper clarified liquid was collected to measure the light transmittance of the sample. The light transmittance of the samples was measured at 550 nm, with distilled water serving as the blank control. The assay was performed in triplicate for each wine sample.

Color analysis of the Cornus–kiwifruit wine was conducted using the CIELab color space, employing a Ci7600 colorimeter for testing (X-Rite, Grand Rapids, MI, USA) [46]. The color parameters L*, a*, and b* were utilized to describe the three-dimensional color space. This technique was repeated three times for each sample.

### 2.6. Sensory Evaluation

In accordance with the experimental guidelines in The Science of Wine Tasting, a panel of 16 trained assessors (8 men and 8 women) conducted sensory evaluations of the Cornus–kiwifruit wines. Blind tasting was conducted using ISO-standard wine tasting glasses [47], with samples presented in standardized vessels labeled with randomized three-digit codes. A standardized four-dimensional sensory evaluation system was employed, with the following scoring metrics: visual appearance (20 points), olfactory attributes (30 points), taste profile (30 points), and overall quality (20 points). The raw scores were then converted into a weighted average, expressed on a 10-point scale [48].

### 2.7. Statistical Analysis

The statistical dataset was subjected to a one-way analysis of variance (ANOVA) conducted using IBM SPSS Statistics 27 (IBM Corp., Armonk, NY, USA), with a significance level set at *p* < 0.05.OriginLab 9.1 (Origin Lab Corporation, Northampton, MA, USA) and Omicshare was adopted for image rendering. The results are presented as the mean ± standard deviation (SD) of the three replicates.

## 3. Results

### 3.1. Effects of Various Treatments on Basic Physicochemical Indexes of Cornus–Kiwifruit Wine

**Figure 1:**
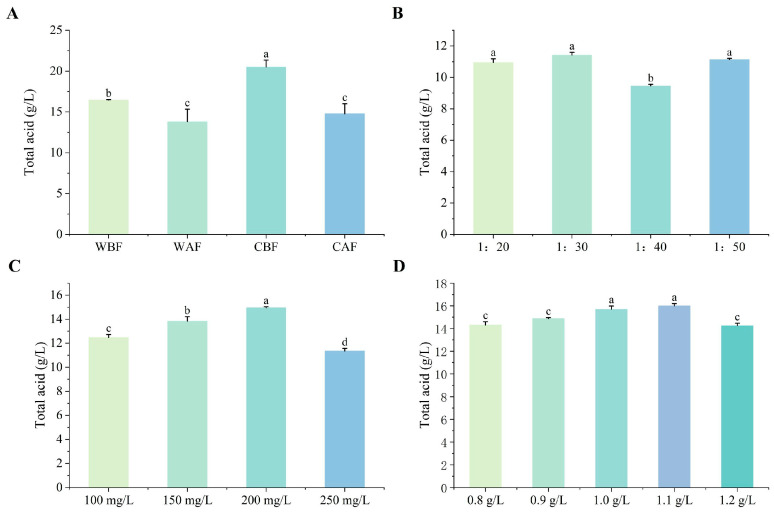
Effects of different treatments on the total acidity of Cornus–kiwifruit wine. (**A**) Addition methods and timing of Cornus officinalis. (**B**) Addition ratios of Cornus officinalis to kiwifruit. (**C**) Amount of yeast additive. (**D**) Different amounts of bentonite additive. Note: WBF = adding whole Cornus officinalis before fermentation, WAF = adding whole Cornus officinalis after fermentation, CBF = adding crushed Cornus officinalis before fermentation, CAF = adding crushed Cornus officinalis after fermentation. The error bars represent the standard deviation of the mean (n = 3). Different letters indicate significant differences among treatments, as determined by one-way ANOVA (*p* < 0.05). The same below.

**Figure 2:**
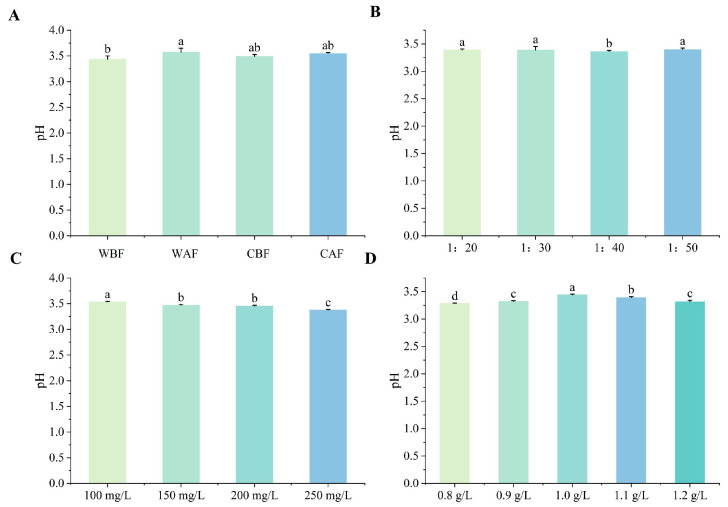
Effects of various treatments on the pH of Cornus–kiwifruit wine. (**A**) Addition methods and timing of Cornus officinalis. (**B**) Addition ratios of Cornus officinalis to kiwifruit. (**C**) Amount of yeast additive. (**D**) Different amounts of bentonite additive.

The total acid content of the composite wine with Cornus officinalis added before fermentation was significantly higher than that of the wine with Cornus officinalis added after fermentation. Furthermore, crushing significantly increased the total acid content in the wine compared to using whole Cornus officinalis (Figure 1A). Notably, there were differences in the total acid content of the wine based on varying Cornus officinalis to kiwi ratios. When the ratio was 1:30, the total acid content was at its highest, while the differences between 1:20 and 1:50 were minimal (Figure 1B). At a yeast supplemental level of 200 mg/L, the total acid content in the wine reached its maximum (Figure 1C). When the bentonite addition was between 1.0 g/L and 1.1 g/L, the total acid content was significantly higher than that of other treatments, with the highest total acid content observed under the 1.1 g/L treatment (Figure 1D). Treatment variations had little effect on pH, as the differences were not significant (Figure 2). The fermentation duration of the whole Cornus officinalis fruit method was 1 day longer than that of crushed fermentation, and the fermentation period for Cornus officinalis added before fermentation was longer than that of Cornus officinalis added after fermentation (Figure S1).

**Figure 3:**
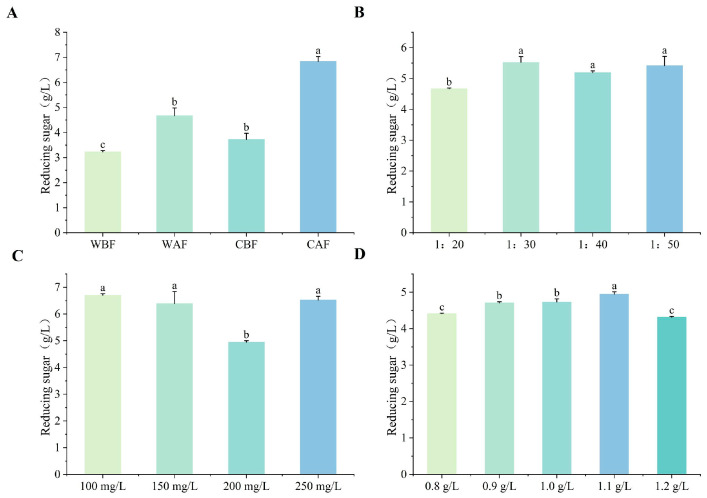
Effects of different treatments on the reducing sugar of Cornus–kiwifruit wine. (**A**) Addition methods and timing of Cornus officinalis. (**B**) Addition ratios of Cornus officinalis to kiwifruit. (**C**) Amount of yeast additive. (**D**) Different amounts of bentonite additive.

The reducing sugar content in wine with Cornus officinalis added after fermentation was higher than that in wine with Cornus officinalis added before fermentation. Additionally, the reducing sugar content of fruit wine produced from crushed Cornus officinalis was higher than that obtained from the addition of whole Cornus officinalis (Figure 3A). The reducing sugar content of Cornus–kiwifruit wine was influenced by different ratios of Cornus officinalis to kiwi fruit used (Figure 3B). The reducing sugar content in the wine reached its minimum level when the yeast additive concentration was 200 mg/L. The other treatment groups with different yeast supplementation levels showed relatively small variations in reducing sugar content, all of which were significantly higher than that of the 200 mg/L treatment group (Figure 3C). When the bentonite addition was 1.1 g/L, the reducing sugar content in the wine was significantly higher than that of other treatments (Figure 3D).

**Figure 4:**
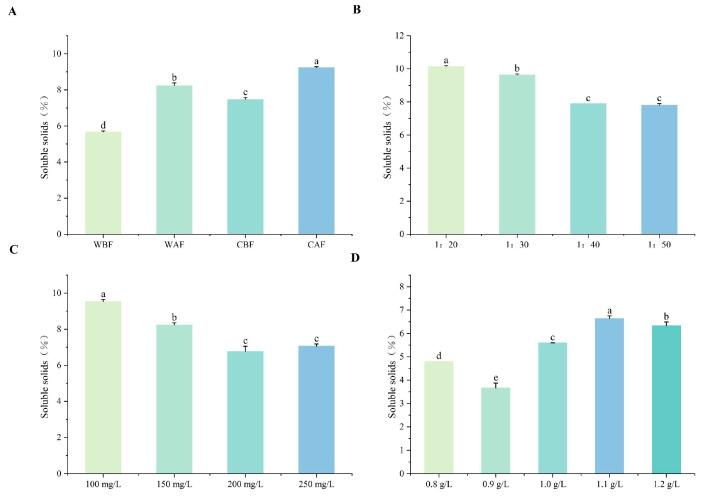
Effects of different treatments on the soluble solids of Cornus–kiwifruit wine. (**A**) Addition methods and timing of Cornus officinalis. (**B**) Addition ratios of Cornus officinalis to kiwifruit. (**C**) Amount of yeast additive. (**D**) Different amounts of bentonite additive.

The soluble solids content in wine can be increased by adding crushed Cornus officinalis and by incorporating crushed Cornus officinalis after fermentation (Figure 4A). A decrease in added Cornus officinalis corresponds to reduced soluble solid content. Notably, the soluble solid content of the wine showed little variation when the Cornus officinalis to kiwi ratios were between 1:40 and 1:50 (Figure 4B). Additionally, when the yeast supplementation level increased from 100 mg/L to 200 mg/L, the soluble solid content initially decreased and then increased, with the lowest content recorded at 200 mg/L (Figure 4C). The wine exhibited the highest soluble solid content at a bentonite concentration of 1.1 g/L, while the lowest content was recorded at 0.9 g/L of bentonite addition (Figure 4D).

**Figure 5:**
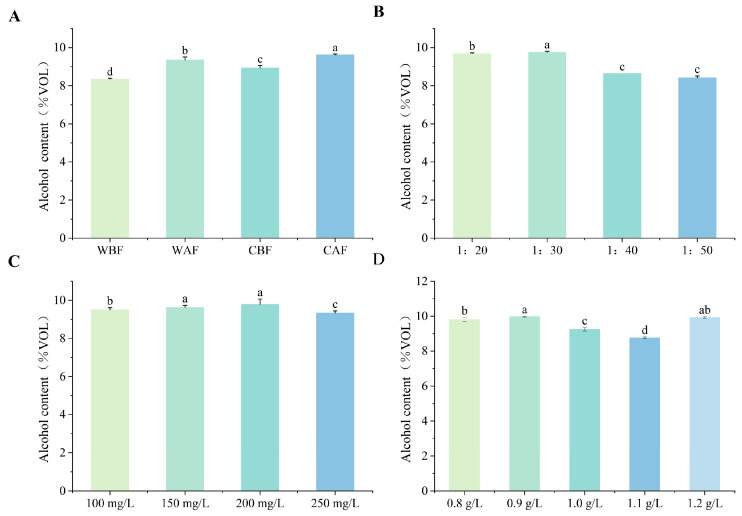
Effects of various treatments on the alcohol content of Cornus–kiwifruit wine. (**A**) Addition methods and timing of Cornus officinalis. (**B**) Addition ratios of Cornus officinalis to kiwifruit. (**C**) Amount of yeast additive. (**D**) Different amounts of bentonite additive.

The alcohol content of wine with Cornus officinalis added before fermentation was significantly lower than that of wine with Cornus officinalis added after (Figure 5A). Different Cornus officinalis to kiwi ratios influenced the alcohol content of the wine. When the ratio was 1:30, the alcohol content reached its maximum. Furthermore, the difference in alcohol content between material ratios of 1:40 and 1:50 was minimal (Figure 5B). When the yeast contents were 150 mg/L and 200 mg/L, the alcohol content reached its maximum (Figure 5C), which correlated well with the observed reduction in sugar content, indicating the efficient conversion of reducing sugars into ethanol. Alcohol content was minimized by a bentonite addition of 1.1 g/L, while treatments with 0.9 g/L and 1.2 g/L yielded significantly elevated alcohol levels relative to other dosages (Figure 5D).

### 3.2. Effects of Various Treatment Methods on Polyphenolic Substances and Unique Components in Cornus-Kiwifruit Wine

The total iridoid glycoside, anthocyanin, flavonoid, and phenol levels in crushed Cornus officinalis composite fruit wine were higher than those in whole Cornus officinalis composite fruit wine (Table 1). Furthermore, adding Cornus officinalis after fermentation enhanced the concentrations of total flavonoids, phenols, anthocyanins, and iridoid glycosides. The total anthocyanin, flavonoid, phenol, and iridoid glycoside levels in the wine significantly decreased with a reduction in Cornus officinalis. In addition, the total iridoid glycoside, anthocyanin, and phenol concentrations in the wine increased with an increase in yeast, reaching the maximum at 200 mg/L and subsequently decreasing. When the yeast addition levels were between 200 mg/L and 250 mg/L, the total anthocyanins, flavonoids, phenolics, and iridoid glycosides in the Cornus–kiwifruit wine were significantly higher. The highest total anthocyanin, phenolics, and iridoid glycoside levels were observed at a yeast addition of 200 mg/L, while the highest total flavonoid content was achieved at 250 mg/L. Total anthocyanin, flavonoid, phenolics, and iridoid glycoside contents in the wine were significantly higher in treatments with bentonite dosages of 1.0, 1.1, and 1.2 g/L than in those with 0.8 and 0.9 g/L. Of these, the 1.1 g/L bentonite group exhibited the highest total flavonoid, phenolic, and iridoid glycoside levels. Furthermore, the highest total anthocyanin content was observed with bentonite supplementation at 1.0 g/L.

### 3.3. Different Treatments on Volatile Compounds in Cornus–Kiwifruit Wine

Volatile compounds in the Cornus–kiwifruit wine derived from different treatments were then identified and quantified (Table 2). A total of 45 volatile compounds were detected, including esters, aldehydes, ketones, terpenes, and alcohols. Ester content, as well as aldehydes and ketones, represented the majority of the volatile compounds, accounting for between 23.17–41.87% and 25.25–40.63%, respectively, with higher levels observed for isoamyl acetate, hexyl acetate, ethyl acetate, hexanal, (E)-2-hexenal, and several others. The analysis identified ten ester compounds, thirteen aldehyde and ketone compounds, ten alcohol compounds, and ten terpene compounds.

Based on our calculations, 25 substances were identified with OAVs > 1 that directly contribute to the aroma profile, as detailed in Table S2. Among these, eight belong to the ester class, including isoamyl acetate, hexyl acetate, ethyl hexanoate, and ethyl acetate. Isoamyl acetate imparts banana and cantaloupe aromas, and hexyl acetate contributes apple and banana scents. Additionally, ten aldehyde and ketone compounds were identified, including (E)-2-hexenal, hexanal, nonanal, and β-Damascone, which confer apple and green grass aromas. Furthermore, five terpenes were detected, including geraniol, β-myrcene, and p-cymene, contributing floral and green herbaceous notes.

The volatile compound contents varied across different Cornus–kiwifruit wine treatments, with some volatile substances exhibiting regular changes. The levels of many volatile compounds in the wine obtained from crushed Cornus officinalis were significantly higher than those derived from whole Cornus officinalis. These included esters such as ethyl acetate and ethyl butyrate; acetophenone, along with other aldehydes and ketones; alcohols like 1-octanol and 1-nonanol; and terpene compounds such as geraniol. The volatile profiles of the wine also differed based on Cornus officinalis to kiwi ratios. Specifically, hexyl acetate and other compounds decreased as the proportion of Cornus officinalis decreased. Conversely, the contents of ethyl acetate and other compounds were the highest at a 1:50 Cornus officinalis-to-kiwi ratio. Additionally, when the yeast addition concentration is 200 mg/L, the proportion of volatile substances such as esters, aldehydes, ketones, and terpenes is high. An analysis of the effects of different bentonite concentrations on the volatile aroma compounds in the wine revealed that the highest aromatic substance contents were obtained at bentonite dosages of 1.0 g/L, 1.1 g/L, and 1.2 g/L. Notably, the 1.1 g/L treatment resulted in significantly higher concentrations of most odor-active compounds than the 1.0 g/L and 1.2 g/L treatments, including isoamyl acetate, ethyl hexanoate, ethyl acetate, (E)-2-hexenal, hexanal, and geraniol.

### 3.4. Effects of Different Treatments on the Color and Clarity of Cornus–Kiwifruit Wine

**Figure 6:**
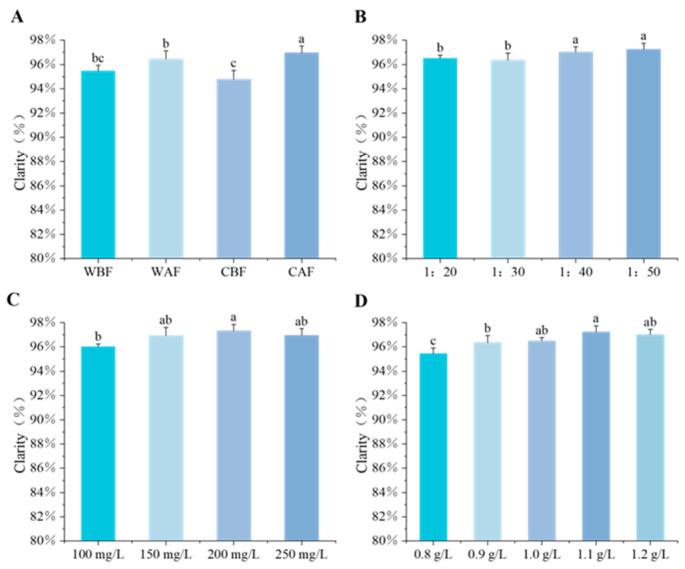
Effects of various treatments on the clarity of Cornus–kiwifruit wine. (**A**) Addition methods and timing of Cornus officinalis. (**B**) Addition ratios of Cornus officinalis to kiwifruit. (**C**) Amount of yeast additive. (**D**) Bentonite additive amount.

The clarity of the Cornus–kiwifruit wine was greater when the addition occurred post-fermentation (Figure 6A), improving with decreasing ratios of 1:20, 1:30, 1:40, and 1:50 (Figure 6B). Initially, as the amount of yeast increased, the clarity also improved, reaching maximum clarity at a yeast addition of 200 mg/L before gradually decreasing (Figure 6C). During the clarification process, increasing the amount of bentonite from 0.8 g/L to 1.2 g/L resulted in varying degrees of clarification. The most significant level of clarity was observed at a bentonite addition of 1.1 g/L (Figure 6D).

**Figure 7:**
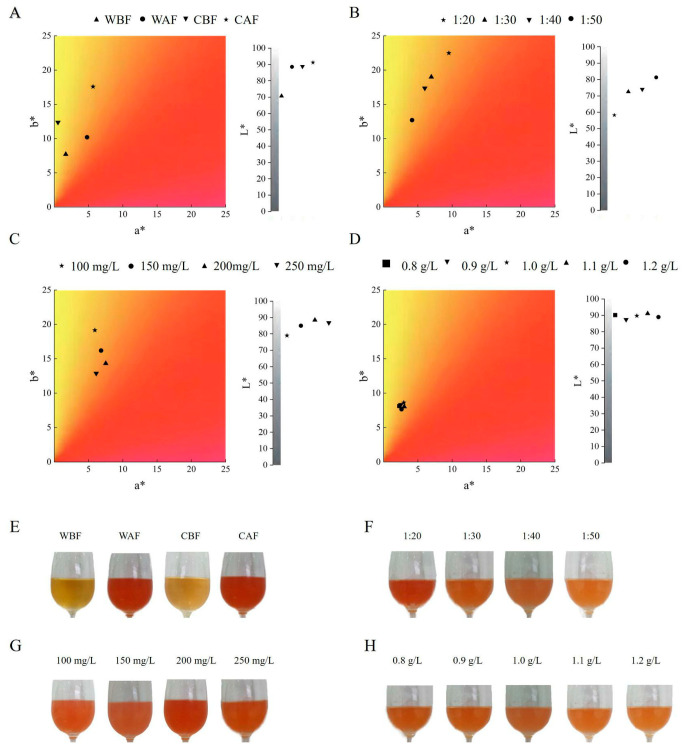
Effects of different treatments on the color of Cornus–kiwifruit wine. (**A**) Addition methods and timing of Cornus officinalis. (**B**) Addition ratios of Cornus officinalis to kiwifruit. (**C**) Yeast additive amount. (**D**) Amount of bentonite additive. (**E**) Addition methods and timing of Cornus officinalis. (**F**) Addition ratios of Cornus officinalis to kiwifruit. (**G**) Yeast additive amount. (**H**) Amount of bentonite additive.

The a*, b*, and L* values for Cornus–kiwifruit wine produced by adding Cornus officinalis after fermentation were significantly higher than those obtained by adding it before fermentation (Figure 7A). The color of the wine made through the post-fermentation addition of Cornus officinalis was garnet red, while the color of the wine produced through the pre-fermentation addition was straw yellow (Figure 7E). The a* and b* values decreased significantly with material ratios of 1:20, 1:30, and 1:40 (Figure 7B). Consequently, the wine’s color transitioned from garnet red to amber (Figure 7F). As yeast addition increased from 150 mg/L to 250 mg/L, the a* and L* values initially increased and then decreased, reaching their maximum at 200 mg/L of yeast addition. At this optimal yeast dosage, the wine exhibited the highest red color and brightness (Figure 7C), resulting in a change from garnet red to amber (Figure 7G). Additionally, the b* value showed a declining trend, indicating a decrease in yellow color (Figure 7C). During the clarification process, the a* value in the wine first increased and then decreased as the amount of bentonite added increased from 0.8 g/L to 1.1 g/L, achieving maximum values when the dosage was 1.1 g/L. The b* value followed a similar trend, reaching its maximum at 1.0 g/L (Figure 7D). The color of the wine was amber, although slight variations were observed (Figure 7H).

### 3.5. Comprehensive Quality Analysis of Cornus–Kiwifruit Wine Based on Principal Component Analysis

Principal component analysis (PCA) was performed based on 53 measured indicators, including fundamental physicochemical parameters, phenolic compounds, total iridoid glycosides, and volatile compounds.

**Figure 8:**
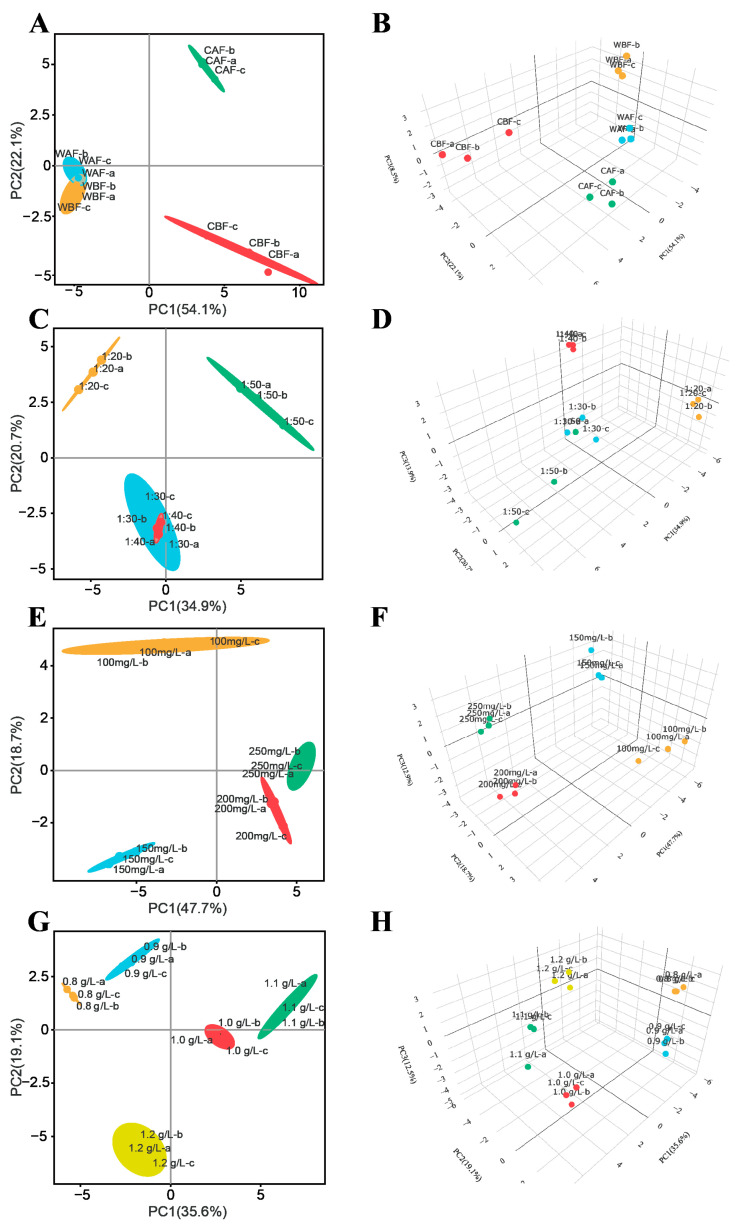
Principal component analysis (PCA) of physicochemical parameters and volatile aroma compounds in Cornus–kiwifruit wine. (**A**) The PCA plot of addition methods and timing in Cornus–kiwifruit wine. (**B**) The 3D PCA plot of addition methods and timing in Cornus–kiwifruit wine. (**C**) The PCA plot of addition ratios of Cornus officinalis to kiwifruit in Cornus–kiwifruit wine. (**D**) The 3D PCA plot of addition ratios of Cornus officinalis to kiwifruit in Cornus–kiwifruit wine. (**E**) The PCA plot of yeast additive amount in Cornus–kiwifruit wine. (**F**) The 3D PCA plot of yeast additive amount in Cornus–kiwifruit wine. (**G**) The PCA plot of bentonite additive amount in Cornus–kiwifruit wine. (**H**) The 3D PCA plot of bentonite additive amount in Cornus–kiwifruit wine.

An analysis of the different Cornus officinalis addition times and methods revealed that PC1 accounted for 54.1% of the total variance, while PC2 and PC3 explained 22.1% and 8.5% (Figure 8A,B), respectively. Alcohols, aldehydes, and ketones, such as 1-octen-3-ol, octanal, nonanal, 3-methyl-1-butanol, benzaldehyde, and β-damascone, contributed significantly along the positive axis of PC1. The crushed and whole-grain treatments were distinctly separated along the PC1 and PC3 axes. Esters (e.g., methyl octanoate and hexyl acetate), total iridoid glycosides, and phenolic compounds exhibited high contributions along the positive PC2 axis, whereas rose oxide terpenoids displayed higher loadings on the negative PC2 axis. The PC3 axis effectively differentiated the treatments based on the addition time.

Among the various ratios of Cornus officinalis and kiwi, PCA demonstrated that PC1, PC2, and PC3 explained 34.9%, 20.7%, and 13.9% of the total variance, respectively (Figure 8C,D). Alcohols, aldehydes, and ketones (e.g., 3-methyl-1-butanol, 1-nonanol, and octanal) showed prominent contributions along the positive PC1 axis, whereas esters (e.g., ethyl caprylate, and ethyl butyrate), total iridoid glycosides, and phenolic compounds exhibited higher loadings on the negative PC1 axis, clearly differentiating between the 1:20 and 1:50 ratios of Cornus officinalis to *Actinidia* sp. Esters and carbonyl compounds (e.g., ethyl hexanoate, hexyl acetate, nonanal, and β-damascone) contributed markedly to the positive PC2 axis, while (Z)-rose oxide and other terpenoids were more prominent on the negative PC2 axis. A clear distinction was observed between the 1:20 and 1:30 treatments, whereas the 1:30 and 1:40 ratios exhibited an overlapping distribution in quadrants III and IV. Except for the 1:40 treatment, which was positioned on the positive PC3 axis, all other ratios were located on the negative PC3 axis.

When analyzing different yeast treatments, the PCA results indicated that PC1, PC2, and PC3 explained 47.7%, 18.7%, and 12.9% of the total variance, respectively (Figure 8E,F). Total iridoid glycosides and phenolic compounds contributed significantly to the positive PC1 axis, whereas most volatile aroma compounds were concentrated on the negative PC1 axis, enabling a clear separation between the 100, 150, and 200 mg/L yeast treatments. The 250 mg/L yeast addition exhibited an overlapping distribution between quadrants III and IV, while a distinct separation was achieved in a 3D-PCA plot for treatments with varying yeast concentrations.

Finally, the PCA of different bentonite addition levels showed that PC1, PC2, and PC3 accounted for 35.6%, 19.1%, and 12.5% of the total variance, respectively (Figure 8G,H). A clear separation was observed between different bentonite treatments in both the PCA and 3D-PCA plots. Basic physicochemical parameters, total iridoid glycosides, phenolic compounds, and most volatile aroma compounds exhibited strong associations with the positive PC1 axis, with 1.1 g/L bentonite displaying the highest value on this axis. Aldehydes and alcohols were primarily associated with the positive PC2 axis, while esters were concentrated on the negative PC2 axis.

The PCA results demonstrate significant differences in the parameters of Cornus officinalis–*Actinidia chinensis* wine across different treatments, thereby imparting distinct characteristics to each group.

### 3.6. Effect of Different Treatments on the Sensory Quality of Cornus–Kiwifruit Wine

**Figure 9:**
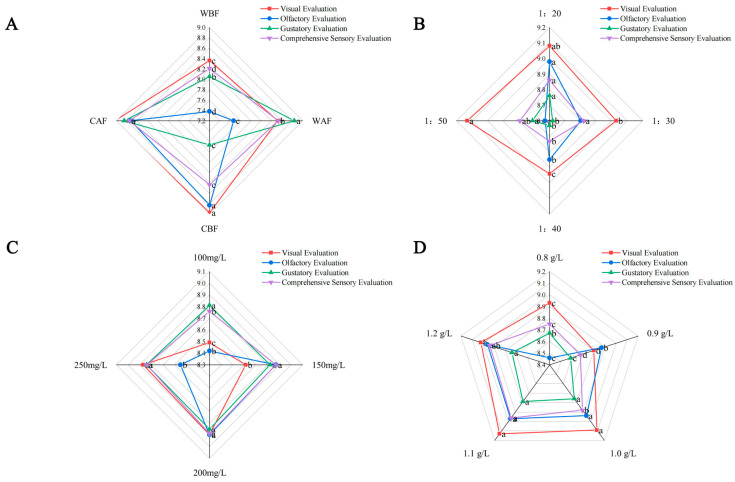
Radar chart depicting sensory evaluation results of Cornus–kiwifruit wine. (**A**) Addition methods and timing of Cornus officinalis. (**B**) Addition ratios of Cornus officinalis to kiwifruit. (**C**) Amount of yeast additive. (**D**) Different amounts of bentonite additive.

The sensory evaluation results were systematically analyzed using radar plots (Figure 9). Significant differences were observed in sensory attributes among various processing methods. Cornus–kiwifruit wines produced with crushed Cornus officinalis exhibited superior visual and olfactory characteristics compared to those containing whole berries. Notably, pre-fermentation addition resulted in significantly lower gustatory scores than post-fermentation addition, likely attributable to the perceived inferior mouthfeel associated with higher acidity and lower sugar content. The CAF treatment demonstrated the highest overall sensory score (Figure 9A). Regarding fruit ratio optimization, wines prepared with Cornus officinalis to kiwi ratios of 1:20 and 1:50 showed significantly enhanced performance in visual appearance, taste perception, and comprehensive evaluation. The 1:20 ratio achieved the maximum olfactory rating, while the 1:50 combination yielded the lowest overall score (Figure 9B). Yeast inoculation levels exhibited a minimal impact on gustatory perception, and the 200 mg/L addition significantly improved visual appeal, aromatic profile, and total sensory assessment (Figure 9C). The bentonite concentration substantially influenced wine quality, with the 1.0, 1.1, and 1.2 g/L treatments demonstrating significantly elevated scores in visual, olfactory, gustatory, and overall evaluations compared to other concentrations. The 1.1 g/L treatment consistently achieved the highest sensory ratings across all of the assessed parameters (Figure 9D).

## 4. Discussion

### 4.1. Effects of Different Treatments on Basic Physicochemical Indices of Cornus–Kiwifruit Wine

In the fermentation of fruit wine, yeast plays a significant role in determining quality. Selecting an appropriate yeast with excellent fermentation capabilities is advantageous for enhancing the quality of kiwi wine and reducing its alcohol content [49]. Adding 200 mg/L of yeast optimizes the fermentation rate and ensures complete fermentation in Cornus–kiwifruit wine, yielding the lowest reducing sugar and highest alcohol content. This is likely due to insufficient yeast activity at lower doses, causing incomplete fermentation, high residual sugar, and low alcohol yield. Conversely, higher yeast doses may deplete sugars excessively, reducing ethanol production and increasing residual yeast [50].

The results indicate that Cornus officinalis addition significantly modulates ethanol and total acidity in Cornus–kiwifruit wine [51]. Post-fermentation supplementation yielded elevated ethanol content and total acidity compared with pre-fermentation incorporation, and the total acid content of Cornus–kiwifruit wine was significantly elevated compared with that of kiwifruit wine alone [52]. Furthermore, composite wines prepared with crushed Cornus officinalis consistently demonstrated superior total acid content over those incorporating whole berries. This enhanced acidity likely stems from the intrinsic acidic properties of Cornus officinalis, which potentiate the baseline acidity of the wine. The crushed form may optimize substrate contact and maceration efficiency during processing [53].

The flavor and color of wine are influenced by various treatment methods during fermentation and clarification. For example, research indicates that different types of clarifying agents produce distinct effects on the color of mulberry wine [54], with some clarifiers outperforming mixed clarifiers [55]. The clarity and brightness of Cornus–kiwi wine in our study were maximized with the addition of 1.1 g/L bentonite, resulting in improved color performance. Furthermore, using appropriate yeast to initiate fermentation is crucial for maintaining the color stability of fruit wine [56]. blueberry wines undergoing shorter fermentation times and higher fermentation temperatures exhibit more red and brown tones [57], and in this study, higher yeast concentrations decreased the Cornus–kiwifruit wine’s yellow hue. The highest red hue was observed at 200 mg/L, where clarity was also maximized, suggesting that yeast residues from excessive dosing may reduce clarity [16]. Consequently, the selection and quantity of yeast and clarifying agents can be optimized through further research to produce high-quality composite fruit wines. The composite wines produced with post-fermentation Cornus officinalis addition exhibited significantly better color and clarity than those with pre-fermentation addition. Higher Cornus officinalis concentrations reduced clarity and decreased red color intensity, suggesting an inhibitory effect on clarification. The red color is a characteristic attribute of Cornus officinalis, contributing to the distinctive appearance of Cornus–kiwifruit wine. This finding is consistent with earlier studies indicating that Cornus officinalis wines tend to develop sedimentation during storage, often resulting in turbidity and precipitation that substantially compromise product quality [58]. Therefore, optimizing clarification techniques is critical for quality control. Future studies should investigate not only dosage effects, but also the efficacy of different clarifying agents and their synergistic combinations in improving Cornus–kiwifruit wine clarity.

### 4.2. Effects of Different Adding Methods of Cornus officinalis on Polyphenolic Substances and Special Components in Cornus–Kiwifruit Wine

The phenolic compounds in composite fruit wine are influenced by various factors, including the timing of addition and soaking duration, which affect the active health components of phenols. For instance, the soaking time affects both the active ingredients and antioxidant capacity of wolfberry wine [59]. In addition, when herbs are added after fermentation, both phenolic compounds and antioxidant activities significantly increase compared to the additions before fermentation [60]. In our study, adding Cornus officinalis after fermentation enhanced the extraction of polyphenolic substances in Cornus–kiwifruit wine, and crushing the fruit increased its polyphenolic substances. This effect may stem from two interrelated mechanisms: the naturally high phenolic concentration in Cornus officinalis berries and improved extraction efficiency achieved through mechanical processing. The crushing process increases the surface area exposure of fruit tissues during maceration, thereby facilitating the more effective dissolution and transfer of phenolic compounds into the must. These findings suggest that optimized physical disruption of fruit tissues represents a critical processing parameter for maximizing the bioactive potential of Cornus officinalis wines [61].

Adding Cornus officinalis to kiwifruit wine not only enhances polyphenolic content, but also introduces iridoids and iridoid glycosides, which are characteristic bioactive compounds of the fruit responsible for its health benefits. Ethanol-soluble iridoids reach higher concentrations in wines than in fresh fruits [62], resulting in more potent hepatoprotective, nephroprotective, and hypoglycemic effects than those of the raw material [63]. With the evolution of consumer consumption attitudes, preferences have increasingly shifted toward the nutritional, health-promoting, and functional benefits of fruit wines. Both polyphenols and iridoid glycosides increase with higher Cornus officinalis concentrations and improved crushing methods, likely due to enhanced extraction efficiency. Notably, winemaking processing modifies the acidic profile of Cornus officinalis while simultaneously enhancing both iridoid glycoside content (typically low in traditional preparations) and sensory quality. Although wine and mead processing effectively increases principal iridoid glycosides and improves flavor characteristics, the underlying extraction and transformation mechanisms require further investigation [64].

### 4.3. Effects of Different Addition Methods on Aroma Compound Content in Cornus–Kiwifruit Wine

The aroma of fruit wine is a key factor in its quality evaluation, reflecting not only flavor characteristics, but also influencing consumers’ overall perceptions. The evaluation of aroma is primarily related to the content and complexity of aroma compounds. Kiwi wine produced through mixed fermentation with various yeast strains exhibits an increased concentration of volatile compounds, thereby enhancing its floral and fruity notes [65]. Additionally, different yeast inoculation methods significantly affect the quality and aromatic profile of these wines [66], and their aroma and taste can be significantly improved by identifying and screening yeast [67]. Indeed, previous studies significantly emphasized the selection of yeast. Nevertheless, the quantity of yeast added at the onset of fermentation plays a crucial role in determining the aroma profile of Cornus–kiwifruit wine, and this study reveals that varying yeast inoculation levels significantly influence the concentration of volatile aroma compounds. The 150 mg/L and 200 mg/L yeast additions exhibited higher concentrations of most volatile aroma compounds, while the 100 mg/L and 250 mg/L treatments showed lower levels. This phenomenon may be due to insufficient fermentation caused by an inadequate yeast quantity at 100 mg/L, resulting in incomplete aroma development and potential negative impacts from yeast autolysis byproducts at 250 mg/L that could compromise Cornus–kiwifruit wine aroma quality [68].

Adding specific substances to fruit wine can alter the characteristics of aroma compounds, enhance flavor, and improve quality. For instance, incorporating reduced glutathione into cider [69], assimilable nitrogen [70] and ammonium salts [71] into wine, and active ingredient extracts [72] into health-oriented wine can modify the content of volatile substances and aroma characteristics, thus enhancing flavor and improving quality. This study found incorporating Cornus officinalis introduced a unique class of compounds—iridoid glycosides—into the kiwifruit wine. Crushed Cornus officinalis added both pre- and post-fermentation addition significantly increased the concentration of aromatic compounds in the wine, with the resulting samples performing well in sensory evaluation. The Cornus–kiwifruit wine not only developed aromatic compounds during fermentation, but also typically synthesized aging-related aroma compounds during maturation. It is hypothesized that Cornus officinalis, particularly in its crushed form, may facilitate the formation of these aging-related aromas [73].

Kiwifruits demonstrate significant maturity variation within the orchard, with variability observed even within individual canopies. To ensure consistent maturity levels, phased harvesting strategies are often employed [74]. Differences in the basic physicochemical parameters, phenolic content, and volatile aroma compounds were observed among the four groups of Cornus–kiwifruit wine. These variations may be attributed to differences in harvest timing. Such temporal variations could influence fruit quality parameters such as firmness and aromatic profile [75,76], consequently affecting the final wine quality. Within the same treatment group, as the kiwifruits were collected concurrently with same quality parameters, the variations detected among Cornus–kiwifruit wines could be predominantly attributed to differential processing conditions, which demonstrated the treatment’s effects on wine quality characteristics.

## 5. Conclusions

Cornus officinalis introduced iridoid glycosides to the kiwi wine. The total acid, phenols, iridoid glycosides, and volatile aroma substances significantly increased when the fruit of Cornus officinalis was crushed and added after fermentation. Furthermore, the amount of Cornus officinalis influenced the wine’s quality. An increase in the Cornus officinalis-to-kiwifruit ratio enhanced the levels of volatile aroma compounds, phenolic compounds, and other active health-promoting ingredients, such as iridoid glycosides and hexyl acetate. However, the excessive use of Cornus officinalis can negatively affect clarity. The composite wine produced with a yeast addition of 200 mg/L exhibited the highest concentrations of phenolic compounds and total iridoid glycosides, along with the highest relative volatile aromatic compound content, including esters, aldehydes, ketones, and terpenes. Furthermore, the sensory evaluation results for this sample were significantly superior to those of the other experimental groups. The concentrations of phenolic compounds, total iridoid glycosides, and the majority of volatile aromatic compounds were notably higher with bentonite addition levels of 1.0 g/L, 1.1 g/L, and 1.2 g/L. Additionally, these samples exhibited superior clarity and color characteristics. Comparative analysis indicated that the 1.1 g/L bentonite treatment yielded Cornus–kiwifruit wine with significantly enhanced physicochemical and sensory properties compared to the other experimental groups, accompanied by the highest overall sensory evaluation scores. Thus, this research provides a theoretical foundation for the production of high-quality Cornus–kiwi composite fruit wine.

## Figures and Tables

**Table 1:** Effects of different treatments on the contents of polyphenolic substances and special components in Cornus–kiwifruit wine (mg/L).

	Treatments		Total Anthocyanins	Total Flavonoids	Total Phenolics	Total Iridoid Glycosides
Cornus officinalis added in different ways and times	1	WBF	1.53 ± 0.10 d	274.24 ± 19.17 d	676.47 ± 20.21 d	2591.75 ± 88.84 d
	2	WAF	6.90 ± 0.28 b	433.01 ± 8.36 b	1317.62 ± 49.31 b	4353.99 ± 23.62 b
	3	CBF	2.99 ± 0.44 c	322.82 ± 3.14 c	777.30 ± 33.59 c	3760.86 ± 120.59 c
	4	CAF	10.14 ± 0.52 a	670.90 ± 7.43 a	1462.55 ± 37.80 a	5761.85 ± 20.41 a
Different ratio of Cornus officinalis and kiwi	5	1:20	18.49 ± 1.08 a	575.38 ± 6.47 a	1406.65 ± 69.03 a	4486.49 ± 210.64 a
	6	1:30	17.41 ± 0.46 a	553.13 ± 27.05 a	1357.76 ± 32.17 a	3897.45 ± 121.90 b
	7	1:40	7.89 ± 1.69 b	507.92 ± 11.08 b	1321.77 ± 31.10 a	3246.98 ± 245.47 c
	8	1:50	5.30 ± 0.52 c	417.68 ± 22.46 c	1018.51 ± 59.44 b	2655.78 ± 329.68 d
Different yeast additions	9	100 mg/L	6.27 ± 0.66 b	438.90 ± 15.56 d	1339.87 ± 48.70 b	3716.39 ± 390.30 c
	10	150 mg/L	7.31 ± 1.08 b	494.91 ± 16.60 c	1432.42 ± 10.48 ab	4829.95 ± 117.85 b
	11	200 mg/L	10.13 ± 0.74 a	561.66 ± 14.88 b	1500.12 ± 77.85 a	5648.48 ± 197.32 a
	12	250 mg/L	9.02 ± 0.23 a	641.76 ± 11.79 a	1459.44 ± 83.62 ab	5237.21 ± 138.66 ab
Different amounts of bentonite additive	13	0.8 g/L	2.95 ± 0.16 c	408.29 ± 14.38 c	897.13 ± 24.15 d	2604.05 ± 108.22 b
	14	0.9 g/L	2.85 ± 0.12 c	487.44 ± 9.86 b	996.34 ± 24.60 c	2615.81 ± 59.55 ab
	15	1.0 g/L	4.60 ± 0.39 a	499.44 ± 10.87 b	1174.31 ± 21.66 b	2894.50 ± 38.24 b
	16	1.1 g/L	3.89 ± 0.12 b	520.02 ± 3.05 a	1425.69 ± 41.98 a	3114.52 ± 73.03 a
	17	1.2 g/L	3.86 ± 0.06 b	496.26 ± 6.47 b	918.18 ± 17.23 d	2723.22 ± 56.12 c

Note: WBF = adding whole Cornus officinalis before fermentation, WAF = adding whole Cornus officinalis after fermentation, CBF = adding crushed Cornus officinalis before fermentation, CAF = adding crushed Cornus officinalis after fermentation. The data for each treatment are expressed as the mean ± standard deviation of three replicates. Different lowercase letters within the same group signify statistical significance as determined by one-way ANOVA (*p* < 0.05), and the same letters in the table indicate no significant differences in the number of fungi among treatment groups. The same applies below.

**Table 2:** Effects of different treatments on the contents of volatile substances in Cornus–kiwifruit wine (µg/L).

Aroma Substance	*Cornus officinalis* Added in Different Ways and Times				Different Ratio of *Cornus officinalis* and kiwi				Different Yeast Additions				Different Amounts of Bentonite Additive				
	1	2	3	4	5	6	7	8	9	10	11	12	13	14	15	16	17
	WBF	WAF	CBF	CAF	1:20	1:30	1:40	1:50	100 mg/L	150 mg/L	200 mg/L	250 mg/L	0.8 g/L	0.9 g/L	1.0 g/L	1.1 g/L	1.2 g/L
Esters																	
Methyl Octanoate	24.26 ± 0.06 b	24.20 ± 0.09 b	24.06 ± 0.15 b	26.28 ± 0.41 a	26.22 ± 0.03 a	25.40 ± 0.23 b	25.40 ± 0.04 c	23.66 ± 0.016 d	26.20 ± 0.056 a	26.28 ± 0.40 a	24.83 ± 0.07 b	24.75 ± 0.08 b	24.28 ± 0.14 ab	24.55 ± 0.09 b	24.57 ± 0.40 b	25.08 ± 0.12 a	24.16 ± 0.03 c
Ethyl Caprylate	18.68 ± 0.00 b	38.61 ± 3.57 b	125.22 ± 27.78 a	126.45 ± 14.80 a	113.08 ± 3.02 a	78.20 ± 2.24 b	111.51 ± 3.01 a	67.46 ± 2.93 c	132.34 ± 4.78 a	126.45 ± 14.80 a	69.80 ± 0.29 b	60.28 ± 1.32 b	118.92 ± 1.76 c	124.31 ± 3.00 b	125.15 ± 1.87 b	134.04 ± 2.99 a	128.29 ± 1.90 b
Ethyl Butyrate	46.80 ± 0.54 b	62.95 ± 15.20 b	120.30 ± 27.76 a	149.16 ± 25.22 a	123.67 ± 3.09 a	57.41 ± 1.65 b	45.46 ± 4.48 c	47.20 ± 1.91 c	164.27 ± 3.61 a	159.16 ± 11.08 a	89.56 ± 2.59 b	84.91 ± 0.67 b	115.46 ± 1.99 b	119.12 ± 2.53 b	127.60 ± 3.32 a	129.90 ± 3.19 a	128.62 ± 1.98 a
Isoamyl Acetate	650.27 ± 48.51 b	570.39 ± 58.25 b	1382.80 ± 99.87 a	1485.68 ± 99.25 a	1577.76 ± 82.24 a	694.38 ± 25.48 c	709.35 ± 3.09 c	954.41 ± 3.42 b	1139.58 ± 54.35 a	1185.68 ± 42.16 a	863.82 ± 26.58 a	759.52 ± 18.96 a	1076.59 ± 28.26 d	1012.41 ± 15.52 e	1238.56 ± 24.45 c	1374.69 ± 24.15 a	1310.71 ± 8.17 b
Ethyl Hexanoate	215.13 ± 3.57 b	258.71 ± 2.49 b	684.02 ± 13.79 a	707.13 ± 36.88 a	309.25 ± 14.35 a	223.33 ± 6.75 c	276.14 ± 0.64 b	311.82 ± 7.83 a	439.67 ± 59.73 a	507.13 ± 36.88 a	319.32 ± 7.95 b	261.20 ± 19.78 b	437.74 ± 13.75 d	583.95 ± 10.02 c	690.75 ± 5.90 b	731.25 ± 8.04 a	701.13 ± 3.28 b
Hexyl Acetate	48.13 ± 1.25 c	42.49 ± 10.15 c	153.48 ± 34.61 b	215.05 ± 13.27 a	324.41 ± 14.14 a	143.03 ± 13.03 b	130.76 ± 3.82 b	126.89 ± 7.07 b	179.28 ± 13.10 b	208.27 ± 3.67 a	103.39 ± 0.85 c	64.66 ± 4.34 d	260.69 ± 9.56 b	283.41 ± 6.56 a	260.24 ± 3.40 b	262.40 ± 9.00 b	265.46 ± 2.73 b
Butyl Acrylate	7.64 ± 0.08 a	7.70 ± 0.02 a	7.65 ± 0.08 a	7.63 ± 0.03 a	7.71 ± 0.00 a	7.76 ± 0.06 a	7.66 ± 0.09 a	7.66 ± 0.10 a	7.60 ± 0.00 b	7.63 ± 0.039 a	7.58 ± 0.00 b	7.67 ± 0.00 a	7.67 ± 0.08 a	7.62 ± 0.04 a	7.70 ± 0.01 a	7.72 ± 0.05 a	7.65 ± 0.05 a
Methyl Aalicylate	11.46 ± 0.96 b	11.11 ± 0.48 b	14.67 ± 0.54 a	11.47 ± 0.96 b	11.43 ± 0.95 a	11.37 ± 11.37 a	11.73 ± 1.36 a	10.23 ± 0.75 a	11.84 ± 0.41 a	11.46 ± 0.97 a	11.52 ± 0.06 a	10.76 ± 0.00 a	10.77 ± 0.00 c	11.28 ± 0.43 ab	11.77 ± 0.01 a	11.27 ± 0.45 ab	10.78 ± 0.01 c
Ethyl Acetate	499.04 ± 15.28 b	275.43 ± 18.68 c	514.88 ± 76.82 a	665.13 ± 1.98 a	338.33 ± 11.81 b	333.46 ± 27.24 b	298.61 ± 9.39 b	446.79 ± 22.66 a	470.91 ± 18.12 b	654.68 ± 16.75 a	460.93 ± 1.91 b	671.55 ± 12.18 a	328.26 ± 2.01 c	388.91 ± 4.95 a	365.12 ± 4.47 b	393.23 ± 3.30 a	294.29 ± 2.36 d
Methyl Benzoate	11.04 ± 0.00 b	11.29 ± 0.01 b	11.96 ± 0.19 a	11.26 ± 0.30 b	11.14 ± 0.14 a	11.18 ± 0.18 a	11.33 ± 0.17 a	11.05 ± 0.01 a	11.35 ± 0.11 a	11.16 ± 0.16 a	11.13 ± 0.12 a	11.04 ± 0.00 a	11.29 ± 0.20 a	11.05 ± 0.00 a	11.04 ± 0.00 a	11.20 ± 0.26 a	11.22 ± 0.15 a
Aldehyde ketone																	
(E)-2-Hexenal	532.71 ± 2.87 c	533.16 ± 12.81 c	2304.27 ± 69.93 a	1638.57 ± 76.44 b	773.16 ± 29.29 a	558.28 ± 1.04 c	612.78 ± 10.09 b	579.78 ± 21.87 c	1060.75 ± 33.61 a	792.95 ± 0.45 b	766.691 ± 47.34 b	500.27 ± 24.94 c	538.26 ± 4.30 c	600.91 ± 7.03 b	699.78 ± 5.90 a	707.92 ± 3.95 a	440.07 ± 6.36 d
(E,E)-2,4-Hexadienal	19.45 ± 0.05 b	19.41 ± 0.05 b	23.40 ± 1.24 a	20.97 ± 1.05 b	19.23 ± 0.02 a	19.28 ± 0.08 a	19.26 ± 0.01 a	19.39 ± 0.13 a	19.36 ± 0.11 a	19.92 ± 0.43 a	19.59 ± 0.15 a	19.76 ± 0.68 a	19.30 ± 0.08 a	19.35 ± 0.09 a	19.39 ± 0.14 a	19.35 ± 0.22 a	19.26 ± 0.04 a
Hexanal	947.08 ± 3.18 c	595.21 ± 36.57 a	1929.25 ± 46.75 a	1530.70 ± 96.65 b	940.51 ± 13.41 a	950.44 ± 39.33 a	425.64 ± 15.82 c	835.05 ± 10.67 b	977.16 ± 7.50 b	1530.708 ± 96.65 a	858.38 ± 42.33 c	825.02 ± 15.57 c	1554.53 ± 3.20 c	1673.37 ± 11.10 b	1641.14 ± 25.80 b	1860.08 ± 43.20 a	951.24 ± 43.04 d
Octanal	17.71 ± 0.65 b	16.69 ± 2.32 b	33.64 ± 3.89 a	20.06 ± 1.20 b	13.141 ± 1.92 a	12.98 ± 1.03 a	13.52 ± 1.59 a	17.08 ± 2.14 a	18.56 ± 2.62 a	20.66 ± 0.34 a	18.26 ± 0.71 a	14.67 ± 0.32 b	17.75 ± 0.61 a	14.24 ± 0.60 b	12.42 ± 0.78 c	18.55 ± 0.85 a	18.27 ± 1.49 a
Nonanal	244.59 ± 34.86 d	318.66 ± 13.35 c	633.02 ± 24.29 a	471.11 ± 5.66 b	215.00 ± 6.58 a	190.20 ± 9.08 b	185.02 ± 6.36 b	216.02 ± 8.63 a	345.67 ± 7.34 c	463.76 ± 4.73 a	396.19 ± 11.75 b	244.51 ± 5.48 d	330.68 ± 3.75 c	318.63 ± 2.45 d	309.11 ± 2.09 e	362.34 ± 4.10 a	341.76 ± 1.37 b
(E)-2-Octenal	34.21 ± 3.32 c	29.96 ± 6.48 c	79.15 ± 0.22 b	121.25 ± 20.52 a	30.16 ± 1.56 b	31.57 ± 5.39 b	29.38 ± 0.65 b	45.76 ± 5.51 a	47.63 ± 0.72 a	47.53 ± 2.50 a	42.99 ± 0.31 b	38.58 ± 0.04 c	75.15 ± 1.99 ab	68.87 ± 2.49 c	77.10 ± 2.27 ab	78.84 ± 3.06 a	72.14 ± 3.09 bc
(E,E)-2,4-Heptadienal	5.88 ± 0.02 a	6.03 ± 0.04 a	7.40 ± 2.0 a	6.42 ± 0.14 a	5.97 ± 0.17 a	5.75 ± 0.09 a	6.04 ± 0.13 a	5.77 ± 0.12 a	5.87 ± 0.02 b	6.42 ± 0.14 a	5.77 ± 0.23 b	5.89 ± 0.10 b	5.78 ± 0.16 a	5.76 ± 0.09 a	5.65 ± 0.02 a	5.77 ± 0.07 a	5.69 ± 0.02 a
Benzaldehyde	45.96 ± 0.97 b	43.90 ± 1.55 b	78.21 ± 14.29 a	69.39 ± 0.87 a	32.43 ± 1.98 a	17.07 ± 2.53 b	28.92 ± 0.19 a	15.60 ± 0.46 b	38.34 ± 4.81 b	69.39 ± 0.87 a	36.38 ± 0.02 b	38.82 ± 0.29 b	32.73 ± 1.86 c	44.88 ± 1.76 b	45.39 ± 0.91 b	57.30 ± 1.79 a	55.36 ± 0.13 a
(E,E)-2,4-Nonadienal	15.84 ± 0.01 a	15.98 ± 0.18 a	17.83 ± 2.68 a	15.87 ± 0.00 a	15.84 ± 0.00 a	15.97 ± 0.18 a	15.83 ± 0.01 a	16.09 ± 0.35 a	15.86 ± 0.03 b	15.87 ± 0.00 b	16.27 ± 0.03 a	15.85 ± 0.02 b	15.85 ± 0.02 a	15.88 ± 0.05 a	15.93 ± 0.12 a	15.86 ± 0.03 a	15.84 ± 0.01 a
1-Octen-3-one	9.89 ± 0.19 b	9.49 ± 0.21 b	17.52 ± 1.87 a	10.27 ± 0.07 b	10.13 ± 0.13 a	9.54 ± 0.31 a	9.98 ± 0.19 a	10.87 ± 1.44 a	9.32 ± 0.01 b	10.27 ± 0.07 a	9.99 ± 0.07 a	9.49 ± 0.24 b	9.53 ± 0.19 a	9.49 ± 0.15 a	9.50 ± 0.06 a	9.58 ± 0.27 a	9.80 ± 0.42 a
Carvone	1.95 ± 0.02 a	1.95 ± 0.00 a	2.00 ± 0.00 a	1.98 ± 0.03 a	1.96 ± 0.01 a	1.95 ± 0.01 a	1.96 ± 0.01 a	1.95 ± 0.00 a	1.96 ± 0.00 a	1.98 ± 0.03 a	1.94 ± 0.01 a	1.96 ± 0.01 a	1.95 ± 0.01 a	1.95 ± 0.01 a	1.94 ± 0.01 a	1.95 ± 0.00 a	1.95 ± 0.00 a
β-Damascone	33.68 ± 2.14 c	35.22 ± 5.75 c	183.20 ± 21.15 a	76.32 ± 3.01 b	64.16 ± 0.91 a	42.59 ± 0.83 b	42.53 ± 13.18 b	65.74 ± 15.69 a	36.11 ± 4.51 b	76.32 ± 3.01 a	43.52 ± 1.42 b	37.167 ± 0.29 b	57.06 ± 1.74 a	54.24 ± 1.62 b	53.20 ± 0.05 b	53.41 ± 0.16 b	53.04 ± 0.43 b
Acetophenone	14.09 ± 0.48 a	13.98 ± 3.39 a	19.45 ± 2.59 a	18.70 ± 1.55 a	15.10 ± 0.41 a	12.91 ± 1.88 b	13.97 ± 1.23 a	11.65 ± 0.09 b	17.82 ± 1.96 a	15.70 ± 5.79 a	11.58 ± 0.00 a	13.68 ± 2.95 a	11.54 ± 0.10 b	11.59 ± 0.01 b	12.75 ± 0.28 a	13.25 ± 1.14 a	11.59 ± 0.00 b
Alcohols																	
(Z)-2-Hexenol	48.66 ± 5.38 b	46.39 ± 5.97 b	79.54 ± 4.48 a	76.05 ± 5.26 a	29.78 ± 4.73 b	32.16 ± 9.38 b	55.14 ± 2.86 a	26.80 ± 3.48 b	42.62 ± 7.55 b	76.04 ± 5.26 a	30.85 ± 0.63 c	23.16 ± 0.85 c	57.61 ± 0.40 d	73.56 ± 2.29 b	75.26 ± 0.86 ab	77.91 ± 1.53 a	63.82 ± 2.27 c
4-methyl-1-Pentanol	16.27 ± 1.78 b	5.68 ± 1.98 c	46.91 ± 1.27 a	52.98 ± 5.29 a	7.55 ± 0.17 b	12.06 ± 1.79 a	9.14 ± 0.13 b	13.08 ± 0.60 a	46.82 ± 4.27 a	53.28 ± 4.86 a	17.68 ± 0.93 b	15.85 ± 0.20 b	33.92 ± 0.68 c	37.23 ± 0.94 b	43.86 ± 1.41 a	42.94 ± 2.45 a	41.18 ± 2.56 a
1-Octen-3-ol	45.80 ± 4.35 b	28.59 ± 0.97 c	147.36 ± 28.90 a	76.95 ± 5.19 b	23.63 ± 0.72 a	18.18 ± 0.13 c	20.40 ± 0.24 b	20.25 ± 0.96 b	26.05 ± 0.76 b	39.93 ± 4.72 a	20.47 ± 3.09 b	24.98 ± 0.39 b	25.23 ± 1.27 b	31.35 ± 2.31 a	32.02 ± 1.02 a	33.65 ± 1.38 a	23.17 ± 1.41 b
2-Ethyl-1-hexanol	9.65 ± 0.27 c	18.10 ± 0.83 b	30.92 ± 5.94 a	33.30 ± 1.17 a	7.07 ± 0.90 b	7.85 ± 1.44 b	10.56 ± 0.71 a	13.275 ± 1.68 a	19.59 ± 1.38 a	14.04 ± 2.22 b	12.62 ± 0.79 c	17.24 ± 0.04 a	26.57 ± 0.88 d	30.46 ± 2.09 c	39.07 ± 1.62 b	44.96 ± 2.62 a	44.69 ± 2.03 a
1-Octanol	42.56 ± 4.16 b	32.39 ± 0.52 c	47.57 ± 5.47 b	81.12 ± 0.74 a	28.94 ± 2.98 a	23.30 ± 1.62 b	24.82 ± 0.76 b	5.85 ± 0.35 c	47.36 ± 2.51 a	50.39 ± 1.77 a	47.40 ± 0.42 a	43.50 ± 2.47 a	53.07 ± 1.48 a	49.54 ± 2.08 ab	47.00 ± 0.55 b	46.18 ± 3.22 b	25.31 ± 1.88 c
1-Nonanol	27.01 ± 4.21 b	31.07 ± 5.28 b	116.02 ± 18.41 a	89.68 ± 10.47 a	20.04 ± 0.95 c	20.49 ± 1.34 c	24.23 ± 1.54 b	32.14 ± 3.06 a	54.33 ± 3.47 a	54.68 ± 3.40 a	33.78 ± 1.92 b	26.30 ± 2.30 b	77.66 ± 1.44 a	68.81 ± 0.93 b	56.68 ± 0.97 c	54.14 ± 3.45 c	50.34 ± 2.23 d
1-Butanol	9.71 ± 0.94 a	7.25 ± 0.07 c	8.78 ± 0.52 b	10.47 ± 0.33 a	7.98 ± 0.48 a	7.99 ± 0.72 a	7.63 ± 0.22 a	8.64 ± 0.02 a	7.92 ± 0.39 b	10.47 ± 0.33 a	8.51 ± 0.37 b	8.24 ± 0.60 b	6.93 ± 0.09 bc	7.45 ± 0.33 b	6.68 ± 0.35 c	8.17 ± 0.30 a	6.96 ± 0.21 bc
3-methyl-1-Butanol	859.86 ± 42.49 c	711.01 ± 8.99 c	2585.19 ± 326.18 a	1938.93 ± 127.23 b	759.40 ± 11.32 b	686.56 ± 14.91 c	672.19 ± 6.88 c	939.57 ± 10.90 a	1490.82 ± 41.95 b	1988.93 ± 56.52 a	993.98 ± 8.69 c	788.99 ± 11.23 d	1653.48 ± 36.81 a	787.85 ± 6.02 d	1429.07 ± 17.20 c	1683.45 ± 7.85 a	1544.21 ± 22.97 b
(Z)-3-Hexen-1-ol	10.34 ± 0.34 b	10.13 ± 0.77 b	26.76 ± 4.78 a	23.57 ± 1.43 a	8.97 ± 1.05 b	9.07 ± 1.44 b	7.96 ± 1.20 b	15.69 ± 2.69 a	16.94 ± 5.14 a	23.57 ± 1.43 a	12.46 ± 1.47 b	9.68 ± 0.60 b	16.45 ± 0.19 a	13.71 ± 0.17 b	10.34 ± 0.13 c	16.23 ± 0.96 a	10.68 ± 0.11 c
Phenylethyl Alcohol	464.60 ± 32.80 c	522.30 ± 7.63 c	985.02 ± 45.46 b	1097.05 ± 8.41 a	594.29 ± 1.03 a	446.67 ± 9.16 b	595.99 ± 20.01 a	536.77 ± 24.94 a	805.01 ± 5.84 b	1047.05 ± 62.29 a	473.29 ± 16.75 c	431.59 ± 20.38 c	969.61 ± 7.48 b	590.39 ± 7.58 e	952.94 ± 7.49 c	1051.62 ± 10.94 a	690.18 ± 6.82 d
Terpene																	
β-Myrcene	22.69 ± 0.01 a	22.58 ± 0.01 a	22.66 ± 0.03 a	22.73 ± 0.20 a	22.63 ± 0.05 a	22.66 ± 0.10 a	22.60 ± 0.01 a	23.67 ± 1.54 a	22.81 ± 0.20 a	22.73 ± 0.20 a	22.65 ± 0.09 a	22.63 ± 0.07 a	22.59 ± 0.01 a	22.58 ± 0.01 a	22.60 ± 0.05 a	22.66 ± 0.07 a	22.64 ± 0.09 a
α-Phellandrene	22.64 ± 0.09 a	22.67 ± 0.02 a	22.76 ± 0.01 a	22.73 ± 0.00 a	22.68 ± 0.02 a	22.73 ± 0.06 a	22.68 ± 0.01 a	22.68 ± 0.02 a	22.65 ± 0.02 a	22.73 ± 0.00 a	22.64 ± 0.09 a	22.69 ± 0.02 a	22.69 ± 0.02 ab	22.70 ± 0.03 ab	22.70 ± 0.02 ab	22.72 ± 0.02 a	22.67 ± 0.02 b
D-Limonene	22.73 ± 0.17 b	23.05 ± 0.04 b	23.17 ± 0.08 a	23.40 ± 0.17 a	22.74 ± 0.24 a	23.31 ± 0.50 a	22.97 ± 0.18 a	22.62 ± 0.79 a	23.26 ± 0.94 a	23.40 ± 0.17 a	23.05 ± 0.30 a	22.75 ± 0.32 a	22.93 ± 0.14 b	22.99 ± 0.11 ab	22.85 ± 0.05 b	23.24 ± 0.16 a	22.76 ± 0.19 b
β-Caryophyllene	25.97 ± 0.01 b	25.94 ± 0.01 b	28.05 ± 0.88 a	26.02 ± 0.08 b	25.94 ± 0.03 a	25.98 ± 0.08 a	25.97 ± 0.07 a	27.03 ± 1.49 a	26.00 ± 0.10 a	26.02 ± 0.08 a	26.03 ± 0.06 a	25.96 ± 0.04 a	25.96 ± 0.07 a	25.93 ± 0.01 a	25.96 ± 0.05 a	25.96 ± 0.04 a	25.95 ± 0.03 a
(Z)-Rose Oxide	5.57 ± 0.04 b	5.54 ± 0.01 b	5.89 ± 0.10 a	5.54 ± 0.01 b	5.56 ± 0.04 a	5.62 ± 0.02 a	5.63 ± 0.04 a	5.58 ± 0.05 a	5.66 ± 0.04 a	5.54 ± 0.01 b	5.57 ± 0.04 b	5.61 ± 0.00 a	5.54 ± 0.00 a	5.58 ± 0.07 a	5.54 ± 0.01 a	5.58 ± 0.05 a	5.57 ± 0.05 a
4-Terpinenol	33.03 ± 1.35 b	21.89 ± 0.18 c	78.46 ± 0.69 a	46.66 ± 1.55 b	37.39 ± 4.06 a	23.59 ± 0.97 b	23.89 ± 5.63 b	38.14 ± 4.44 a	45.15 ± 2.69 c	46.66 ± 1.55 c	60.21 ± 3.28 a	52.85 ± 0.09 b	41.09 ± 1.10 b	39.82 ± 1.22 b	41.18 ± 0.41 b	49.80 ± 1.21 a	28.77 ± 2.05 c
α-Terpineol	10.63 ± 0.00 a	11.46 ± 0.44 a	13.37 ± 3.73 a	10.70 ± 0.01 a	14.26 ± 5.13 a	10.09 ± 0.74 a	11.03 ± 0.55 a	10.74 ± 0.13 a	10.68 ± 0.03 a	10.70 ± 0.02 a	10.64 ± 0.02 a	10.67 ± 0.035 a	10.65 ± 0.03 c	11.63 ± 0.01 b	12.61 ± 0.12 a	12.73 ± 0.23 a	10.50 ± 0.23 c
Geraniol	90.85 ± 8.47 a	118.80 ± 8.36 a	159.74 ± 18.62 b	245.39 ± 38.02 a	99.41 ± 7.40 a	60.05 ± 3.28 b	55.62 ± 5.86 b	51.98 ± 7.81 b	179.83 ± 2.30 b	213.39 ± 7.22 a	93.83 ± 4.93 c	82.77 ± 2.63 c	172.97 ± 3.14 d	209.41 ± 1.95 b	206.75 ± 2.10 bc	255.17 ± 4.69 a	201.91 ± 2.17 c
p-Cymene	36.12 ± 0.32 b	36.09 ± 0.24 b	37.14 ± 0.27 a	37.15 ± 0.07 a	36.07 ± 0.26 a	36.88 ± 0.85 a	36.25 ± 0.21 a	34.26 ± 2.26 a	36.35 ± 0.87 a	37.15 ± 0.07 a	36.21 ± 0.51 a	36.11 ± 0.35 a	36.16 ± 0.39 a	36.50 ± 0.61 a	36.11 ± 0.31 a	36.78 ± 0.08 a	36.07 ± 0.31 aa
p-Mentha-1,8-dien-9-olen-9-ol	8.03 ± 0.93 b	12.88 ± 3.01 a	10.22 ± 2.12 a	7.32 ± 0.06 b	8.82 ± 0.75 a	7.25 ± 0.37 a	7.60 ± 0.26 a	8.43 ± 0.73 a	9.14 ± 0.16 a	7.32 ± 0.06 b	7.72 ± 0.15 b	7.62 ± 0.39 b	7.37 ± 0.20 c	7.31 ± 0.06 c	9.83 ± 0.16 b	13.42 ± 0.51 a	9.44 ± 0.41 b
Others																	
Butanedioic acid	27.81 ± 1.84 c	46.12 ± 3.60 c	249.36 ± 52.06 a	133.73 ± 5.10 b	149.58 ± 4.27 a	110.04 ± 5.41 b	52.01 ± 0.03 c	20.94 ± 1.94 d	80.40 ± 12.25 b	139.23 ± 2.67 a	77.07 ± 6.62 b	69.43 ± 8.76 b	102.07 ± 1.90 a	104.52 ± 0.95 a	102.11 ± 1.83 a	104.91 ± 0.60 a	104.63 ± 2.57 a
2-Methylpyrazine	16.85 ± 0.45 b	16.83 ± 0.07 b	21.95 ± 3.06 a	17.92 ± 1.51 b	16.73 ± 0.77 a	16.52 ± 0.54 a	15.67 ± 0.29 a	16.88 ± 0.34 a	17.77 ± 1.10 a	17.92 ± 1.51 a	17.36 ± 1.08 a	17.03 ± 0.28 a	16.37 ± 0.20 b	17.50 ± 0.42 a	15.81 ± 0.24 b	17.48 ± 0.36 a	16.20 ± 0.42 b

Note: Concentrations of volatile compounds were quantified by curves of chromatographically pure standards and expressed as the means ± SD (n = 3).

## Data Availability

Data are contained within the article or Supplementary Material.

## Supplementary Materials

The following supporting information can be downloaded at: https://www.mdpi.com/article/10.3390/foods14101705/s1, Figure S1 Effects of different treatments on the fermentation day of Cornus–kiwifruit wine; Table S1 Experimental Methods Summary Table; Table S2 Substances with OAV > 1 and their concentrations.
