# Supplementary material for: Effects of Different Fermentation and Clarification Methods on the Color, Physicochemical Characteristics, and Aroma Profile of Healthcare Cornus–Kiwifruit Composite Wine
Source: Foods. 2025 May 11;14(10):1705. doi: 10.3390/foods14101705 (PMC12111703; doi:10.3390/foods14101705)
Supplement: Supplementary file 1 [file foods-14-01705-s001.zip › foods-3577849-supplementary.pdf]

Supplementary data for

Effects of Different Fermentation and Clarification Methods on the Color, Physicochemical Characteristics, and Aroma Profile of Healthcare Cornus–Kiwifruit Composite Wine

Cuiyan Zeng[a], Xueru Zhang[a], Junxia Zhang[a], Shuiyan Pan[a], Keqin Chen[a*], Yulin Fang[a*].

[a] College of Enology, Northwest A&F University, Yangling, 712100, Shaanxi, China

*** Corresponding authors:**

Keqin Chen, Email: chenkeqin1985@nwafu.edu.cn;

Yulin Fang, Email: fangyulin@nwsuaf.edu.cn;

Present address:

College of Enology, Northwest A&F University, Yangling, 712100, China.

Supplementary Data Captions

Figure S1 Effects of different treatments on the fermentation day of Cornus-kiwifruit wine.

Table S1 Experimental Methods Summary Table.

Table S2 Substances with OAV > 1 and their concentrations.

Figure S1

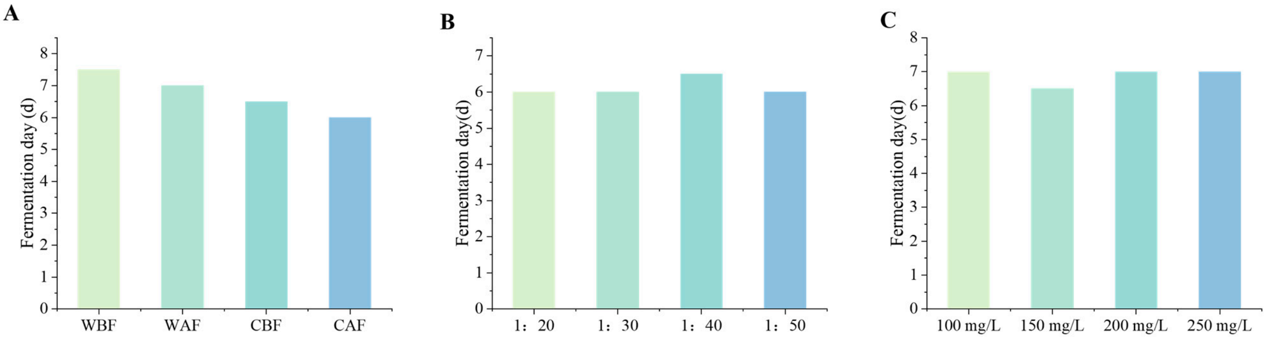

Figure S1 Effects of different treatments on the fermentation day of Cornus-kiwifruit wine.(A) Addition methods and timing of Cornus officanalis. (B) Addition ratios of cornus officinalis to kiwi fruit. (C) Amount of yeast additive.

Note: WBF = adding whole Cornus officinalis before fermentation, WAF = adding whole Cornus officinalis after fermentation, CBF =adding crushed Cornus officinalis before fermentation, CAF =adding crushed Cornus officinalis after fermentation. The error bars represent the standard deviation of the mean (n = 3). Different letters indicate significant differences among treatments, as determined by one-way ANOVA ($p < 0.05$). The same below.

Table S1

Table S1 Factorial design table of experimental treatments.

Numbering	Treatment	Adding method and of Cornus officinalis	ratio of Cornus officinalis and kiwi	Yeast addition level	Bentonite addition level
1<br>2<br>3<br>4	Cornus officinalis added in different ways and times	**WBF**<br>**WAF**<br>**CBF**<br>**CAF**	1:20	200 mg/L	1.1 g/L
5<br>6<br>7<br>8	Different ratio of Cornus officinalis and kiwi	CAF	**1:20**<br>**1:30**<br>**1:40**<br>**1:50**	200 mg/L	1.1 g/L
9<br>10<br>11<br>12	Different yeast additions	CAF	1:20	**100 mg/L**<br>**150 mg/L**<br>**200 mg/L**<br>**250 mg/L**	1.1 g/L
13<br>14<br>15<br>16<br>17	Different amounts of bentonite additive	CAF	1:20	200 mg/L	**0.8 g/L**<br>**0.9 g/L**<br>**1.0 g/L**<br>**1.1 g/L**<br>**1.2g/L**

Note: WBF = adding whole Cornus officinalis before fermentation, WAF = adding whole Cornus officinalis after fermentation, CBF =adding crushed Cornus officinalis before fermentation, CAF =adding crushed Cornus officinalis after fermentation. Variables within each group were presented in bold font in the table.

Table S2

Table S2 Substances with OAV > 1 and their concentrations.

Aroma substance	CAS	Threshold（μg/L）	Cornus officinalis added in different ways and times				Different ratio of Cornus officinalis and kiwi				Different yeast additions				Different amounts of bentonite additive				
			1	2	3	4	5	6	7	8	9	10	11	12	13	14	15	16	17
			WBF	WAF	CBF	CAF	1:20	1:30	1:40	1:50	100mg/L	150mg/L	200mg/L	250mg/L	0.8g/L	0.9g/L	1.0g/L	1.1g/L	1.2g/L
Esters																			
Ethyl Caprylate	106-32-1	19.3	18.68±0.00b	38.61±3.57b	125.22±27.78a	126.45±14.80a	113.08±3.02a	78.20±2.24b	111.51±3.01a	67.46±2.93c	132.34±4.78a	126.45±14.80a	69.80±0.29b	60.28±1.32b	118.92±1.76c	124.31±3.00b	125.15±1.87b	134.04±2.99a	128.29±1.90b
Ethyl Butyrate	105-54-4	9	46.80±0.54b	62.95±15.20b	120.30±27.76a	149.16±25.22a	123.67±3.09a	57.41±1.65b	45.46±4.48c	47.20±1.91c	164.27±3.61a	159.16±11.08a	89.56±2.59b	84.91±0.67b	115.46±1.99b	119.12±2.53b	127.60±3.32a	129.90±3.19a	128.62±1.98a
Isoamyl Acetate	123-92-2	0.15	650.27±48.51b	570.39±58.25b	1382.80±99.87a	1485.68±99.25a	1577.76±82.24a	694.38±25.48c	709.35±3.09c	954.41±3.42b	1139.58±54.35a	1185.68±42.16a	863.82±26.58a	759.52±18.96a	1076.59±28.26d	1012.41±15.52e	1238.56±24.45c	1374.69±24.15a	1310.71±8.17b
Ethyl Hexanoate	123-66-0	5	215.13±3.57b	258.71±2.49b	684.02±13.79a	707.13±36.88a	309.25±14.35a	223.33±6.75c	276.14±0.64b	311.82±7.83a	439.67±59.73a	507.13±36.88a	319.32±7.95b	261.20±19.78b	437.74±13.75d	583.95±10.02c	690.75±5.90b	731.25±8.04a	701.13±3.28b
Hexyl Acetate	142-92-7	115	48.13±1.25c	42.49±10.15c	153.48±34.61b	215.05±13.27a	324.41±14.14a	143.03±13.03b	130.76±3.82b	126.89±7.07b	179.28±13.10b	208.27±3.67a	103.39±0.85c	64.66±4.34d	260.69±9.56b	283.41±6.56a	260.24±3.40b	262.40±9.00b	265.46±2.73b
Butyl Acrylate	141-32-2	2	7.64±0.08a	7.70±0.02a	7.65±0.08a	7.63±0.03a	7.71±0.00a	7.76±0.06a	7.66±0.09a	7.66±0.10a	7.60±0.00b	7.63±0.039a	7.58±0.00b	7.67±0.00a	7.67±0.08a	7.62±0.04a	7.70±0.01a	7.72±0.05a	7.65±0.05a
Ethyl Acetate	141-78-6	5	499.04±15.28b	275.43±18.68c	514.88±76.82a	665.13±1.98a	338.33±11.81b	333.46±27.24b	298.61±9.39b	446.79±22.66a	470.91±18.12b	654.68±16.75a	460.93±1.91b	671.55±12.18a	328.26±2.01c	388.91±4.95a	365.12±4.47b	393.23±3.30a	294.29±2.36d
Methyl Benzoate	93-58-3	7.3	11.04±0.00b	11.29±0.01b	11.96±0.19a	11.26±0.30b	11.14±0.14a	11.18±0.18a	11.33±0.17a	11.05±0.01a	11.35±0.11a	11.16±0.16a	11.13±0.12a	11.04±0.00a	11.29±0.20a	11.05±0.00a	11.04±0.00a	11.20±0.26a	11.22±0.15a
Aldehyde ketone																			
(E)-2-Hexenal	6728-2	398.1	532.71±28.7c	533.16±12.	2304.27±69.93a	1638.57±76.44b	773.16±29.29a	558.28±10.4c	612.78±10.	579.78±21.	1060.75±33.61a	792.95±0.45b	766.91±47.34b	500.27±24.	538.26±4.30c	600.91±7.03b	699.78±5.90a	707.92±3.95a	440.07±6.36d

Aroma substance	CAS	Threshold（μg/L）	Cornus officinalis added in different ways and times				Different ratio of Cornus officinalis and kiwi				Different yeast additions				Different amounts of bentonite additive				
			**1**	**2**	**3**	**4**	**5**	**6**	**7**	**8**	**9**	**10**	**11**	**12**	**13**	**14**	**15**	**16**	**17**
			**WBF**	**WAF**	**CBF**	**CAF**	**1:20**	**1:30**	**1:40**	**1:50**	**100mg/L**	**150mg/L**	**200mg/L**	**250mg/L**	**0.8g/L**	**0.9g/L**	**1.0g/L**	**1.1g/L**	**1.2g/L**
	6-3			81c					09b	87c				94c					
(E,E)-2,4-Hexadienal	142-83-6	2	19.45±0.05b	19.41±0.05b	23.40±1.24a	20.97±1.05b	19.23±0.02a	19.28±0.08a	19.26±0.01a	19.39±0.13a	19.36±0.11a	19.92±0.43a	19.59±0.15a	19.76±0.68a	19.30±0.08a	19.35±0.09a	19.39±0.14a	19.35±0.22a	19.26±0.04a
Hexanal	66-25-1	5	947.08±3.18c	595.21±36.57a	1929.25±46.75a	1530.70±96.65b	940.51±13.41a	950.44±39.33a	425.64±15.82c	835.05±10.67b	977.16±7.50b	1530.78±96.65a	858.38±42.33c	825.02±15.57c	1554.53±3.20c	1673.37±11.10b	1641.14±25.80b	1860.08±43.20a	951.24±43.04d
Octanal	124-13-0	0.587	17.71±0.65b	16.69±2.32b	33.64±3.89a	20.06±1.20b	13.141±1.92a	12.98±1.03a	13.52±1.59a	17.08±2.14a	18.56±2.62a	20.66±0.34a	18.26±0.71a	14.67±0.32b	17.75±0.61a	14.24±0.60b	12.42±0.78c	18.55±0.85a	18.27±1.49a
Nonanal	124-19-6	1.1	244.59±34.86d	318.66±13.35c	633.02±24.29a	471.11±5.66b	215.00±6.58a	190.20±9.08b	185.02±6.36b	216.02±8.63a	345.67±7.34c	463.76±4.73a	396.19±11.75b	244.51±5.48d	330.68±3.75c	318.63±2.45d	309.11±2.09e	362.34±4.10a	341.76±1.37b
(E)-2-Octenal	2548-87-0	3	34.21±3.32c	29.96±6.48c	79.15±0.22b	121.25±20.52a	30.16±1.56b	31.57±5.39b	29.38±0.65b	45.76±5.51a	47.63±0.72a	47.53±2.50a	42.99±0.31b	38.58±0.04c	75.15±1.99ab	68.87±2.49c	77.10±2.27ab	78.84±3.06a	72.14±3.09bc
(E,E)-2,4-Heptadienal	4313-03-5	0.1	5.88±0.02a	6.03±0.04a	7.40±2.0a	6.42±0.14a	5.97±0.17a	5.75±0.09a	6.04±0.13a	5.77±0.12a	5.87±0.02b	6.42±0.14a	5.77±0.23b	5.89±0.10b	5.78±0.16a	5.76±0.09a	5.65±0.02a	5.77±0.07a	5.69±0.02a
(E,E)-2,4-Nonadienal	5910-87-2	0.1	15.84±0.01a	15.98±0.18a	17.83±2.68a	15.87±0.00a	15.84±0.00a	15.97±0.18a	15.83±0.01a	16.09±0.35a	15.86±0.03b	15.87±0.00b	16.27±0.03a	15.85±0.02b	15.85±0.02a	15.88±0.05a	15.93±0.12a	15.86±0.03a	15.84±0.01a
1-Octen-3-one	4312-99-6	0.003	9.89±0.19b	9.49±0.21b	17.52±1.87a	10.27±0.07b	10.13±0.13a	9.54±0.31a	9.98±0.19a	10.87±1.44a	9.32±0.01b	10.27±0.07a	9.99±0.07a	9.49±0.24b	9.53±0.19a	9.49±0.15a	9.50±0.06a	9.58±0.27a	9.80±0.42a
β-Damascone	23726-93-4	0.002	33.68±2.14c	35.22±5.75c	183.20±21.15a	76.32±3.01b	64.16±0.91a	42.59±0.83b	42.53±13.18b	65.74±15.69a	36.11±4.51b	76.32±3.01a	43.52±1.42b	37.167±0.29b	57.06±1.74a	54.24±1.62b	53.20±0.05b	53.41±0.16b	53.04±0.43b
Alcohols																			
1-Oct	339	15	45.80±	28.59±	147.36±	76.95±5.	23.63±0.	18.18±	20.40±	20.25±	26.05±0.	39.93±4.	20.47±3.	24.98±	25.23±1.	31.35±2.	32.02±1.	33.65±1.	23.17±1.

Aroma substance	CAS	Threshold (μg/L)	Cornus officinalis added in different ways and times				Different ratio of Cornus officinalis and kiwi				Different yeast additions				Different amounts of bentonite additive				
			1	2	3	4	5	6	7	8	9	10	11	12	13	14	15	16	17
			WBF	WAF	CBF	CAF	1:20	1:30	1:40	1:50	100mg/L	150mg/L	200mg/L	250mg/L	0.8g/L	0.9g/L	1.0g/L	1.1g/L	1.2g/L
en-3-ol	1-86-4		4.35b	0.97c	28.90a	19b	72a	0.13c	0.24b	0.96b	76b	72a	09b	0.39b	27b	31a	02a	38a	41b
3-methyl-1-Butanol	123-51-3	4	859.86±42.49c	711.01±8.99c	2585.19±326.18a	1938.93±127.23b	759.40±11.32b	686.56±14.91c	672.19±6.88c	939.57±10.90a	1490.82±41.95b	1988.93±56.52a	993.98±8.69c	788.99±11.23d	1653.48±36.81a	787.85±6.02d	1429.07±17.20c	1683.45±7.85a	1544.21±22.97b
Terpene																			
β-Myrcene	123-35-3	1.2	22.69±0.01a	22.58±0.01a	22.66±0.03a	22.73±0.20a	22.63±0.05a	22.66±0.10a	22.60±0.01a	23.67±1.54a	22.81±0.20a	22.73±0.20a	22.65±0.09a	22.63±0.07a	22.59±0.01a	22.58±0.01a	22.60±0.05a	22.66±0.07a	22.64±0.09a
α-Phellandrene	99-83-2	4	22.64±0.09a	22.67±0.02a	22.76±0.01a	22.73±0.00a	22.68±0.02a	22.73±0.06a	22.68±0.01a	22.68±0.02a	22.65±0.02a	22.73±0.00a	22.64±0.09a	22.69±0.02a	22.69±0.02ab	22.70±0.03ab	22.70±0.02ab	22.72±.02a	22.67±0.02b
(Z)-Rose Oxide	16409-43-1	1	5.57±0.04b	5.54±0.01b	5.89±0.10a	5.54±0.01b	5.56±0.04a	5.62±0.02a	5.63±0.04a	5.58±0.05a	5.66±0.04a	5.54±0.01b	5.57±0.04b	5.61±0.00a	5.54±0.00a	5.58±0.07a	5.54±0.01a	5.58±0.05a	5.57±0.05a
Geraniol	106-24-1	6.6	90.85±8.47a	118.80±8.36a	159.74±18.62b	245.39±38.02a	99.41±7.40a	60.05±3.28b	55.62±5.86b	51.98±7.81b	179.83±2.30b	213.39±7.22a	93.83±4.93c	82.77±2.63c	172.97±3.14d	209.41±1.95b	206.75±2.10bc	255.17±4.69a	201.91±2.17c
p-Cymene	99-87-6	5.01	36.12±0.32b	36.09±0.24b	37.14±0.27a	37.15±0.07a	36.07±0.26a	36.88±0.85a	36.25±0.21a	34.26±2.26a	36.35±0.87a	37.15±0.07a	36.21±0.51a	36.11±0.35a	36.16±0.39a	36.50±0.61a	36.11±0.31a	36.78±0.08a	36.07±0.31aa

Note: WBF = adding whole Cornus officinalis before fermentation, WAF = adding whole Cornus officinalis after fermentation, CBF =adding crushed Cornus officinalis before fermentation, CAF =adding crushed Cornus officinalis after fermentation. The data for each treatment is expressed as the mean ± standard deviation of three replicates. Different lowercase letters within the same group signify statistical significance as determined by one-way ANOVA ($p<0.05$). Concentrations of volatile compounds were quantified by curves of chromatographically pure standards and expressed as the means ± SD (n=3).
